# Importance of Multiple-window Assessment for the Diagnosis of Ascending Aortic Dissection Using Point-of-care Ultrasound: Report of Three Cases

**DOI:** 10.5811/cpcem.2019.6.43245

**Published:** 2019-08-05

**Authors:** Virginia Zarama, María C. Arango-Granados, Luis A. Bustamante Cristancho

**Affiliations:** Fundación Valle del Lili, ICESI University, Department of Emergency Medicine, Cali, Colombia

## Abstract

Acute ascending aortic dissection has a high mortality rate and requires rapid diagnosis and treatment. Point-of-care ultrasound (POCUS) can aid in the diagnosis. The aortic root is usually evaluated in the parasternal long-axis view; however, a dissection flap is not always visible in this projection. We present three cases of acute, type A aortic dissection in which the dissection flap was only evident in the apical five-chamber and subxyphoid views. These cases suggest that POCUS may play a pivotal role in the initial diagnosis of acute ascending aortic dissection and highlight the importance of viewing multiple windows to fully evaluate this possibility.

## INTRODUCTION

Acute ascending aortic dissection (AD) is a potentially catastrophic disease associated with high in-hospital mortality (58%). Even with surgical repair, fatality rates are high (26%); therefore rapid recognition, diagnosis, and treatment is warranted.^1^ AD is difficult to diagnose in the emergency department (ED) because it is both a rare clinical condition and one that does not always present in a classic fashion.^2^ Routine initial tests readily available in the ED (e.g., electrocardiogram [ECG], chest radiograph (CXR), and laboratory markers) have variable reliability in making a diagnosis of acute AD. Diagnosis typically involves computed tomography (CT) angiography, transesophageal echocardiography (TEE), or magnetic resonance imaging (MRI).^3^

Point-of-care ultrasound (POCUS) has become an invaluable diagnostic tool in the initial evaluation and management of patients with hemodynamic instability, respiratory insufficiency, or chest pain, and it is safe, rapid, and readily available in many EDs and intensive care units (ICU). A point-of-care focused cardiac ultrasound (FOCUS) study may provide useful information when acute aortic pathology is suspected.^4^ The American Society of Echocardiography (ASE) and the European Association of Cardiovascular Imaging (EACVI) state that the evaluation of the aortic root is best done in the parasternal long-axis view.^5^ However, a dissection flap is not always visible in this projection. Still, the emergency physician must consider this possibility given the significant risk of missing the diagnosis. We present a series of three cases of acute, type-A AD in which the TTE performed at admission revealed a dissection flap only in the apical five-chamber and subxyphoid views.

## CASE REPORTS

### Case 1

**Image 1:**
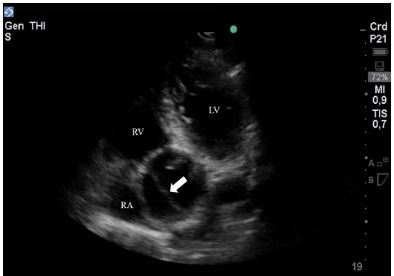
Transthoracic echocardiography apical 5-chamber view of a patient with acute chest pain with a dissection flap (white arrow) visualized in the ascending aorta. *LV*, Left ventricle; *RV*, right ventricle; *RA*, right atrium.

A 63-year-old male with a history of hypertension presented to the ED with four hours of chest pain of moderate intensity. Vital signs were blood pressure (BP) 110/47 milligrams of mercury (mmHg), heart rate (HR) 85 beats per minute (BPM), respiratory rate (RR) 16 breaths per minute (bpm), and oxygen (O_2_) saturation of 99% at room air. The patient was alert, had no signs of pulmonary or systemic congestion, and had normal cardiac sounds without murmurs. The ECG showed inverted T waves in V4, V5, and V6. Immediate medical management was ordered for an acute coronary syndrome. A FOCUS was performed, which showed a normal functioning left ventricle, but a severely dilated aortic root, measuring 6.1 centimeters (cm) in the parasternal long-axis view. No pericardial fluid was identified. An apical five-chamber view was used to visualize the ascending aorta, which clearly showed a dissection flap (Image 1, Video 1).

We suspected an acute, type-A AD, and ordered a TEE to confirm the diagnosis. TEE showed a left ventricular ejection fraction (LVEF) of 60–65%, severe aortic insufficiency and a Stanford type-A AD with the dissection flap at 2.3 cm of the valve plane and extending to the descending aorta. The patient was taken urgently to the operating room for a Bentall procedure. He was then transferred to the ICU and ultimately discharged.

### Case 2

A 64-year-old woman presented with a chief complaint of sudden chest pain, difficulty breathing and collapse. The patient was initially taken to the nearest hospital where she was found in cardiac arrest and received advanced cardiopulmonary resuscitation for 30 minutes, with subsequent return of spontaneous circulation (ROSC). She was immediately transferred to our hospital for post cardiac arrest management and intensive medical care. Upon arrival, the patient was intubated, and her vital signs included a BP of 40/30 mmHg, HR of 53 BPM, RR of 16 bpm and O_2_ saturation of 99% with a fraction of inspired oxygen of 0.5. She had a Glasgow Coma Scale of 6/15, with 3-millimeter (mm) symmetrical and hyporeactive pupils. Heart sounds were regular, rhythmic, with no murmurs, and pulmonary auscultation revealed bilateral rales. After initial medical stabilization in the ED, the patient was transferred to the ICU where she was received by the on-call emergency physician. The FOCUS exam performed at admission to the ICU revealed a hyperdynamic left ventricle, and a normal right ventricle with no signs of ventricular overload; there was no evidence of pericardial fluid, and the inferior vena cava was 1.7 cm with a 50% respiratory collapse.

**CPC-EM Capsule**

What do we already know about this clinical entity?

*Acute ascending aortic dissection is a potentially catastrophic disease. Experts recommend the parasternal long-axis view for ultrasound diagnosis*.

What makes this presentation of disease reportable?

*In these cases the dissection flap was only evident in the apical five-chamber and subxyphoid views*.

What is the major learning point?

*This series highlights the importance of viewing multiple windows to fully evaluate the possibility of acute ascending aortic dissection*.

How might this improve emergency medicine practice?

*Viewing multiple windows may improve the diagnostic accuracy of point-of-care ultrasound to evaluate the possibility of acute aortic dissection*.

**Image 2:**
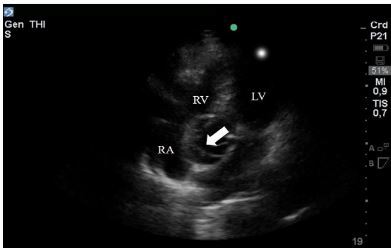
Transthoracic echocardiography apical five-chamber view of a patient in post-cardiac arrest with a dissection flap (white arrow) visualized in the ascending aorta. *LV*, left ventricle; *RV*, right ventricle; *RA*, right atrium.

A dilated aortic root of 4.5 cm was found in the parasternal long-axis view. An apical five-chamber view was used to better visualize the ascending aorta, and a dissection flap was visualized (Image 2, Video 2). We then evaluated the proximal abdominal aorta, which also showed the dissection flap. The patient underwent CT imaging, which confirmed the type-A AD. Unfortunately, she suffered from severe hypoxic encephalopathy due to the prolonged cardiac arrest, and her neurological status did not improve after intensive care medical management. In agreement with the family, the patient was not taken to a surgical repair of the AD, and she died two days after admission.

### Case 3

A 77-year-old woman with no past medical history was found lying unconscious. Two hours prior she had complained of severe pain of no clear anatomic location or characteristics. At arrival to the ED she was found to be in profound shock, with severe bradycardia. Her vital signs included a HR of 30 BPM, BP of 50/30 mmHg and an O_2_ saturation of 60%. Initial management was started with atropine and dopamine infusion, but the patient became pulseless. Advanced cardiac life support commenced, with ROSC two minutes after active chest compressions.

**Image 3:**
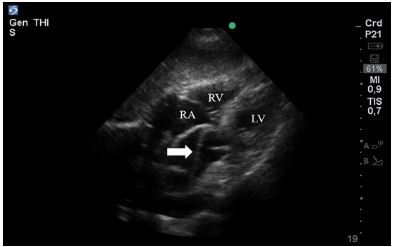
Transthoracic echocardiography subxyphoid view of a patient in post-cardiac arrest with a dissection flap (white arrow)visualized in the ascending aorta. *LV*, left ventricle; *RV*, right ventricle; *RA*, right atrium.

In the immediate post-arrest period, a POCUS was performed. The subxyphoid window revealed a dissection flap in the ascending aorta (Image 3, Video 3), with extension to the abdominal aorta. TEE confirmed the diagnosis of type-A AD with extension to the abdominal aorta. Cardiovascular surgeons decided on emergent surgical repair. However, the patient persisted with severe hypotension despite high vasopressor support and massive transfusion strategies. Further ultrasonographic evaluation revealed free fluid in the splenorenal pouch, which was absent at arrival, and so aortic rupture was suspected. Her condition continued to deteriorate and, unfortunately, the patient died three hours after admission.

## DISCUSSION

Acute AD is an emergent and potentially fatal disorder that can cause complications such as cardiac tamponade, aortic valve insufficiency, or hypoperfusion syndromes. Population-based studies show an incidence of acute dissection from 2 to 3.5 cases per 100,000 person-years.^1^ The most common risk condition for AD is hypertension (present in 75% of the cases). Other risk factors include the following: smoking; direct blunt trauma; the use of illicit drugs (such as cocaine or amphetamines); genetic disorders associated with abnormalities of the aortic wall (i.e., Marfan, Loeys-Dietz, and Ehlers-Danlos syndromes); bicuspid aortic valve; and inflammatory or infectious conditions involving the aorta.^1^ The International Registry of Aortic Dissection reports an overall in-hospital mortality of 27.4%, with the highest mortality among patients managed medically without surgical intervention (58%). Even those patients taken to immediate surgical repair have a mortality of 26%.^1^

The Stanford classification defines type A and type B ADs as those involving and not involving the ascending aorta, respectively. When the dissection involves the ascending aorta, emergency surgical repair is usually indicated, whereas medical therapy is the initial strategy for acute dissections involving only the descending aorta.^2^ AD has a wide range of clinical presentations and may mimic other more common conditions. Patients usually present with chest, back or abdominal pain that is abrupt in onset, severe in intensity, described as ripping or tearing, and can radiate from the chest or back to the abdomen or to the lower extremities. Physical exam is usually unrevealing, but some signs can be encountered such as a pulse deficit, a systolic BP differential between extremities, a focal neurological deficit, a new aortic insufficiency murmur, hypotension, or shock.^1^

ECG changes in AD are usually nonspecific, with 30% of patients showing no abnormalities and 42% showing nonspecific ST-segment and T-wave changes. CXR may show findings suggestive of AD (widening of the mediastinum, widening of the aortic contour, displaced calcification, aortic kinking, and opacification of the aorticopulmonary window) in 88.6% of patients. However, it can be normal in 11.3% of patients; thus, a normal CXR does not exclude AD.^1^

Patients with suspected thoracic AD require early and accurate diagnosis. Aortography has been replaced by less-invasive imaging techniques including TEE, helical CT, and MRI, which have clinically equally reliable diagnostic values for confirming or ruling out thoracic AD with a pooled sensitivity of 98%–100% and specificity of 95%–98%.^6^

TTE is a valuable clinical tool for the assessment of critically ill patients, including those with acute chest pain syndromes where differential diagnoses are broad. The ASE and the ECVI state that the evaluation of the aortic root is best done in the parasternal long-axis view.^5^ A dilated aortic root has a sensitivity and specificity of 77–91% and 72–95%, respectively, for the diagnosis of ascending AD. More importantly, visualization of an ascending AD flap by TTE has specificity as high as 98%, therefore becoming a valuable tool to confirm this condition.^7^–^10^ However, a dissection flap is not always visible in the parasternal long-axis view.

As reported in these three cases, the visualization of multiple windows such as the apical five-chambers and subxyphoid views can sometimes reveal intimal abnormalities otherwise not evident. The former can be obtained as an extension to the apical 4-chamber with anterior tilting of the transducer, and the latter with the probe placed in the subxyphoid space, aimed to the left and angled cephalad toward the thorax. If a TTE is suggestive of AD, the clinical policy from the American College of Emergency Physicians recommends immediate surgical consultation or transfer to a higher level of care (Level C recommendation).^11^

Additionally, POCUS can be useful in assessing high-risk features or complications such as pericardial effusion or severe aortic insufficiency and in diagnosing other serious conditions. This diagnostic tool can be of valuable importance for the differential diagnosis of acute chest pain. As illustrated in Case 1, ECG changes could lead to a misdiagnosis of an acute coronary syndrome. Furthermore, starting anti-aggregation, anticoagulation, or thrombolysis could have potentially fatal effects when the underlying disease is an AD. The prompt diagnosis and timely management of this severe aortic pathology could result in better clinical outcomes and reduced costs of care, although this is yet to be proven. Even so, the use of POCUS for the diagnosis of acute ascending AD is increasing.^12^,^13^

## CONCLUSION

POCUS is a valuable tool in the diagnosis and management of critically ill patients. It is safe, rapid, and readily available in many EDs and ICUs. These three cases suggest that POCUS may play a pivotal role in the initial diagnosis of acute ascending aortic dissection and highlight the importance of viewing multiple windows to fully evaluate this possibility. It is our belief that POCUS should be a part of the initial clinical evaluation of patients with hemodynamic instability, chest pain, or respiratory insufficiency to expedite the diagnostic evaluation and to initiate and guide emergent treatment.

## Supplementary Information

Video 1. Transthoracic echocardiography apical 5-chamber view of a patient with acute chest pain with a dissection flap visualized in the ascending aorta.

Video 2. Transthoracic echocardiographyapical five-chamber view of a patient in post-cardiac arrest with a dissection flap visualized in the ascending aorta.

Video 3. Transthoracic echocardiography subxyphoid view of a patient in post-cardiac arrest with a dissection flap visualized in the ascending aorta.
